# Vaccinia Scars Associated with Improved Survival among Adults in Rural Guinea-Bissau

**DOI:** 10.1371/journal.pone.0000101

**Published:** 2006-12-20

**Authors:** Mette Lundsby Jensen, Sangeeta Dave, Maarten Schim van der Loeff, Carlos da Costa, Tim Vincent, Aleksandra Leligdowicz, Christine Stabell Benn, Adam Roth, Henrik Ravn, Ida Maria Lisse, Hilton Whittle, Peter Aaby

**Affiliations:** 1 The Medical Research Council Laboratories, Fajara, The Gambia; 2 Projecto de Saúde de Bandim, Statens Serum Institut, Bissau, Guinea-Bissau; 3 Department of Pathology, Herlev University Hospital, Copenhagen, Denmark; University of California, San Francisco, United States of America

## Abstract

**Background:**

In urban Guinea-Bissau, adults with a vaccinia scar had better survival but also a higher prevalence of HIV-2 infection. We therefore investigated the association between vaccinia scar and survival and HIV infection in a rural area of Guinea-Bissau.

**Methodology/Principal Findings:**

In connection with a study of HIV in rural Guinea-Bissau, we assessed vaccinia and BCG scars in 193 HIV-1 or HIV-2 infected and 174 uninfected participants. Mortality was assessed after 2½–3 years of follow-up. The analyses were adjusted for age, sex, village, and HIV status. The prevalence of vaccinia scar was associated with age, village, and HIV-2 status but not with sex and schooling. Compared with individuals without any scar, individuals with a vaccinia scar had better survival (mortality rate ratio (MR) = 0.22 (95% CI 0.08–0.61)), the MR being 0.19 (95% CI 0.06–0.57) for women and 0.40 (95% CI 0.04–3.74) for men. Estimates were similar for HIV-2 infected and HIV-1 and HIV-2 uninfected individuals. The HIV-2 prevalence was higher among individuals with a vaccinia scar compared to individuals without a vaccinia scar (RR = 1.57 (95% CI 1.02–2.36)).

**Conclusion:**

The present study supports the hypothesis that vaccinia vaccination may have a non-specific beneficial effect on adult survival.

## Introduction

Vaccinia vaccination was introduced in 1800 and was associated with marked reductions in mortality in the industrialising countries [1]. The last case of smallpox occurred in 1977 [2] and in 1980 the World Health Organization recommended stopping vaccinia vaccinations. No assessment was made of the health impact of discontinuing vaccination.

Vaccines may have non-targeted effects–that is, effects which can not be explained by the prevention of the vaccine targeted infection. Live vaccines, including measles and BCG vaccines [3]–[7] have been associated with reductions in overall mortality which cannot be explained merely by the prevention of measles or tuberculosis infection. Studies have also consistently found that such non-targeted effects are larger for females [3], [7]–[9]. These observations made us study the possible non-targeted effects of vaccinia. A study of 1,893 adults from Guinea-Bissau conducted between 1998 and 2002 showed lower mortality among vaccinia scar positive individuals more than 20 years after the last vaccinia vaccinations. Adults with a smallpox scar had a mortality rate ratio (MR) of 0.60 (0.41–0.87) compared to those without any scar. Also, individuals with a vaccinia scar had larger mid-upper-arm-circumference (MUAC) than individuals without a scar and a larger MUAC was associated with better survival [10].

Scientists from the MRC Laboratories, The Gambia, have examined HIV-2 infection in rural Guinea-Bissau for the last 15 years [11]–[18]. Due to the surprising association between vaccinia scars and survival in urban Bissau, the team was asked to investigate the possible role of vaccinia vaccinations for survival in rural Guinea-Bissau.

## Methods

An HIV-2 study has been conducted in Caio, a rural area in Guinea-Bissau since 1989. Detailed descriptions are provided elsewhere [11], [12], [15]. Briefly, two general population surveys were conducted in 1989–91 and 1997. Following the initial survey, a case-control study examining clinical conditions and immunological parameters was conducted in 1991. Those enrolled in this study were re-examined in 1996 and 2003. The 2003 study round included additional HIV-1 and HIV-2 infected individuals identified in the 1997 population study and a similar number of new controls who were frequency-matched according to sex, age, and village were included. The 2003 study included 402 individuals of whom 145 were HIV-2 single infected, 29 were HIV-1 single infected, 30 were dually infected, and 198 were HIV negative.

HIV-infected and uninfected individuals were invited for a clinical examination conducted by a physician and blood samples were collected for assessment of HIV and HTLV infections as well as for T-cell subsets, malaria, anaemia, and sexually transmitted infections. As part of the clinical examination, the mid upper-arm circumference (MUAC) was measured in mm using a TALC insertion tape (Teaching Aids at Low Cost, St. Albans, England).

During the clinical examination, the physician examined the presence of vaccinia and BCG vaccination scars on fore and upper arms. Scar reading was conducted by 4 medical doctors of whom one (SD) examined 78% of the participants. The physicians had a common protocol for measuring and classifying the scars. The questionnaire had room for noting three scars. If there were more than three scars these would usually include several vaccinia scars and a BCG scar. The physicians were told to register at least one scar of each type, i.e. both vaccinia and BCG. The procedure may have led to underreporting of people with more than two vaccinia scars. The number of scars were therefore only analysed as 2 or more scars versus one vaccinia scar. For each scar the height and width were measured with a ruler. The average of the two diameters was used as an overall index of the size of the scar. Also the location of the scar was marked. Scars were classified as vaccinia, BCG, or “unknown origin”. Based on the experience from Bissau, the protocol specified that vaccinia scars are usually more than 10 mm, have a smooth central area and a rough peripheral rim whereas BCG scars are usually small (<8 mm) and smooth.

Survival was assessed in early 2006. Annual censuses have been conducted between 2003 and 2006. In February 2006, field assistants verified all identified deaths and visited all study participants who had not been seen in a clinical examination conducted in January and February 2006. A few additional deaths were identified as a result of these visits; survival status could be ascertained of all participants

The main ethnic group in Caio is the “Manjaco” which has a traditional age group system; with intervals of around 4 years a new age cohort is established. The group will have a unique name and individuals will belong to this cohort for the rest of their life. In a specific age group, the females are a few years younger than the males. Individuals are socially attached to their group and will remember the name of the group to which they belong. This traditional age group system is likely to reflect relative age better than official chronological age from the colonial era. The birth day is poorly remembered and may be manipulated for reason of tax, military service, or admission to school. Hence, the traditional system is likely to be more accurate than the self-reported chronological age. The majority of the people in the study area were Manjacos but 9 individuals belonged to other groups and were age classified according to the self-reported chronological age. In all analyses, we adjusted for age using age sets consisting of three sequential Manjaco age groups.

### Statistical analysis

The study from urban Bissau revealed that few had received vaccinia vaccination before school-age, the median age of vaccination being 18 years [10]. Since vaccinia vaccinations were stopped in Guinea-Bissau in 1980, we only included individuals born before 1974 in the previous [10] and the present analysis. A Cox proportional hazards model [19] was used to assess the mortality ratio for vaccinated and unvaccinated individuals. The Cox model had follow-up time as underlying time and followed the individuals from the initial assessment of scars and until death or the survey in 2006. Age was controlled using the traditional age group system described above. HIV status was categorised as HIV-1, HIV-2 only, and HIV negative. The association between vaccination scar status and respectively HIV-2 seropositivity and MUAC was analysed by using a general linear model. HIV-1 was not present in Guinea-Bissau when smallpox vaccination was still in use. Hence, we have not assessed the association between scar and HIV-1 infection.

## Results

Of the 402 persons taking part in the study, 367 were born before 1974. Of these, 141 were only HIV-2 infected, 23 were only HIV-1 infected, 29 were dually infected, and 174 were uninfected. The distribution of these 367 individuals on the three types of scar (vaccinia, BCG, unknown) is indicated in Table 1; 68% (N = 251) had a vaccinia scar (Table 1), and of these 58% (146/251) had additionally a BCG or a scar of unknown origin. Of the 116 individuals without a vaccinia scar, 70% (81/116) had a BCG or a scar of unknown origin. A total of 44% (160/367) of the cohort had a BCG scar, and 34% (123/367) had a scar of unknown origin. Ten percent (35/367) had no vaccination scar. The median diameter was 20 mm (25–75 percentiles: 16–24 mm) for vaccinia scars, 10 mm (6–15 mm) for BCG scars, and 8 mm (5–11 mm) for scars of unknown origin. As seen in table 2, vaccinia vaccination is strongly associated with age group. There was no significant difference in the vaccinia scar prevalence for men and women, the prevalence ratio being 1.11 (95% CI 0.97–1.27) controlled for age. The prevalence of vaccinia scar was associated with village (Table 2). No association was found between years of schooling (Table 2) or current alcohol consumption pattern (data not shown) and the presence of vaccinia scar.

**Table 1:**
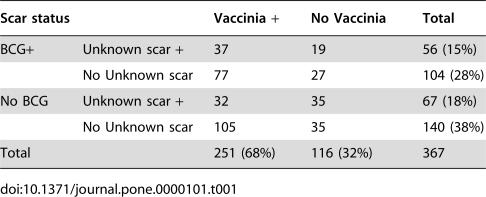
Vaccinia, BCG and unknown scars among 367 residents born before 1974.

Scar status		Vaccinia +	No Vaccinia	Total
BCG+	Unknown scar +	37	19	56 (15%)
	No Unknown scar	77	27	104 (28%)
No BCG	Unknown scar +	32	35	67 (18%)
	No Unknown scar	105	35	140 (38%)
Total		251 (68%)	116 (32%)	367

**Table 2:**
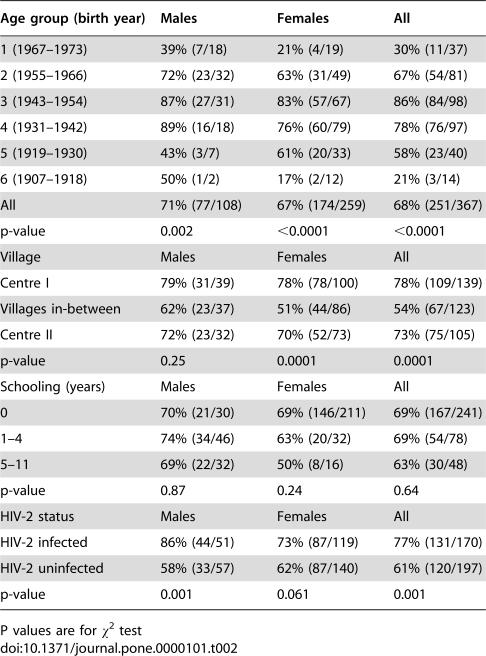
Prevalence of vaccinia scars by sex, age, village, years of schooling, and HIV-2 status.

Age group (birth year)	Males	Females	All
1 (1967–1973)	39% (7/18)	21% (4/19)	30% (11/37)
2 (1955–1966)	72% (23/32)	63% (31/49)	67% (54/81)
3 (1943–1954)	87% (27/31)	83% (57/67)	86% (84/98)
4 (1931–1942)	89% (16/18)	76% (60/79)	78% (76/97)
5 (1919–1930)	43% (3/7)	61% (20/33)	58% (23/40)
6 (1907–1918)	50% (1/2)	17% (2/12)	21% (3/14)
All	71% (77/108)	67% (174/259)	68% (251/367)
p-value	0.002	<0.0001	<0.0001
Village	Males	Females	All
Centre I	79% (31/39)	78% (78/100)	78% (109/139)
Villages in-between	62% (23/37)	51% (44/86)	54% (67/123)
Centre II	72% (23/32)	70% (52/73)	73% (75/105)
p-value	0.25	0.0001	0.0001
Schooling (years)	Males	Females	All
0	70% (21/30)	69% (146/211)	69% (167/241)
1–4	74% (34/46)	63% (20/32)	69% (54/78)
5–11	69% (22/32)	50% (8/16)	63% (30/48)
p-value	0.87	0.24	0.64
HIV-2 status	Males	Females	All
HIV-2 infected	86% (44/51)	73% (87/119)	77% (131/170)
HIV-2 uninfected	58% (33/57)	62% (87/140)	61% (120/197)
p-value	0.001	0.061	0.001

P values are for χ^2^ test

The mean mid-upper-arm-circumference (MUAC) was 284 mm (range 152–390) for individuals with a vaccination scar and 266 mm (182–352) for individuals without any scar (unadjusted, p = 0.009), the difference being highly significant in an analysis adjusting for sex, age and HIV status (p<0.001). The differential effect was found only among women, the estimated difference between vaccinia scar positive women and women without any scar being 21.0 mm (6.4–35.6) unadjusted, and 21.7 mm (7.3–36.2) when adjusted for age-group and HIV status.

Over the three years of follow-up, 13% (47/367) of the individuals in the cohort died (Table 3). Individuals with a vaccinia-scar had lower mortality than individuals without any scar, the crude mortality rate ratio (MR) being 0.41 (0.19–0.86). Adjusting for age, sex, village and HIV status, the MR was 0.22 (95% CI 0.08–0.61)). The MR for women was 0.19 (95% CI 0.06–0.57) and 0.40 (95% CI 0.04–3.74) for men. The tendency was the same comparing individuals with any scar with individuals without any scar, the MR being 0.25 (95% CI 0.10–0.62) (Table 3). Estimates were beneficial for both HIV-2 infected (MR = 0.19 (0.04–0.79)) and HIV-negative individuals (MR = 0.20 (0.06–0.71)), controlling for age, sex and village. Mortality was 16% (18/116) for those without a vaccinia scar, 12% (25/203) for those with one vaccinia scar and 8% (4/48) for those with 2 or 3 scars. Individuals with 2 or more vaccinia scars may had a MR of 0.14 (0.03–0.56), and those with only one vaccinia scar a MR of 0.24 (0.09–0.68) compared with those without any scar.

**Table 3:**
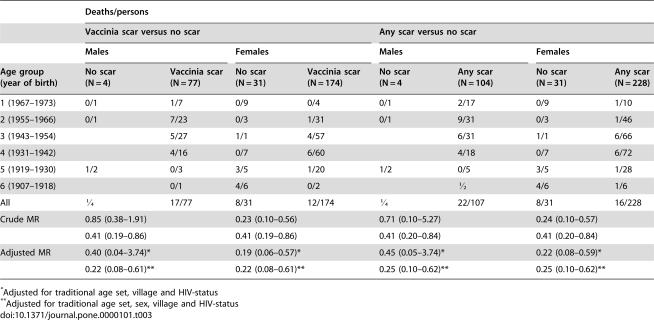
Mortality according to sex, age, and scar status.

	Deaths/persons							
	Vaccinia scar versus no scar				Any scar versus no scar			
	Males		Females		Males		Females	
Age group (year of birth)	No scar (N = 4)	Vaccinia scar (N = 77)	No scar (N = 31)	Vaccinia scar (N = 174)	No scar (N = 4	Any scar (N = 104)	No scar (N = 31)	Any scar (N = 228)
1 (1967–1973)	0/1	1/7	0/9	0/4	0/1	2/17	0/9	1/10
2 (1955–1966)	0/1	7/23	0/3	1/31	0/1	9/31	0/3	1/46
3 (1943–1954)		5/27	1/1	4/57		6/31	1/1	6/66
4 (1931–1942)		4/16	0/7	6/60		4/18	0/7	6/72
5 (1919–1930)	1/2	0/3	3/5	1/20	1/2	0/5	3/5	1/28
6 (1907–1918)		0/1	4/6	0/2		½	4/6	1/6
All	¼	17/77	8/31	12/174	¼	22/107	8/31	16/228
Crude MR	0.85 (0.38–1.91)		0.23 (0.10–0.56)		0.71 (0.10–5.27)		0.24 (0.10–0.57)	
	0.41 (0.19–0.86)		0.41 (0.19–0.86)		0.41 (0.20–0.84)		0.41 (0.20–0.84)	
Adjusted MR	0.40 (0.04–3.74)*		0.19 (0.06–0.57)*		0.45 (0.05–3.74)*		0.22 (0.08–0.59)*	
	0.22 (0.08–0.61)**		0.22 (0.08–0.61)**		0.25 (0.10–0.62)**		0.25 (0.10–0.62)**	

* Adjusted for traditional age set, village and HIV-status

** Adjusted for traditional age set, sex, village and HIV-status

As seen in Table 2, HIV-2 infected individuals were more likely to have a vaccinia scar. In other words, the prevalence of HIV-2 infection was 52% (131/251) among people with a vaccinia scar but only 34% (39/116) among those without. The prevalence ratio (PR) of HIV-2 infection was 1.57 (95% CI 1.14–1.96) controlled for age, sex and village for individuals with a vaccinia-scar compared to those without. The association appeared to be stronger for men (PR = 2.99 (1.25–7.15)) than for women (PR = 1.31 (0.94–1.84)). The prevalence ratio for individuals with a vaccinia scar was 2.08 (95% CI 1.14–3.78) compared with individuals without any scar. A presence of vaccinia scar was not associated with HTLV infection.

## Discussion

The present study was initiated to investigate whether the surprising observations of an association between vaccinia scar and HIV-2 infection and of lower mortality among adults with a vaccinia scar could be repeated. Data collection was independent of data collection in the original study from urban Bissau [10]. The major observations were clearly repeated. The presence of a vaccinia scar was associated with lower mortality and more HIV-2 infection. Additionally, as in Bissau, the beneficial effect of vaccinia scar was strong for women. As found previously, vaccinia scar positive individuals had larger MUACs than scar negative individuals, but only among women. An association with better survival was found for both HIV negative and HIV-2 infected individuals.

Though vaccinia scars are generally larger than BCG scars, there is no exact way to identify whether a scar is due to vaccinia or BCG. The scar classification was made by a physician without knowledge of the participant's HIV-status. The difference in size between vaccinia and BCG scars was similar in Caio and Bissau. However, the measurement of scars in Caio was slightly larger than in previous studies [10], [20], [21] and the prevalence of scars of unknown origin was higher in Caio than in Bissau [10]. Some misclassification is therefore likely and a reexamination of some participants did suggest that some vaccinia scars had been classified as BCG or “unknown”. There was high agreement about positive vaccinia scar readings. It is unlikely that the classification of “no scar” has been differential according to mortality. Hence, misclassification may have led to conservative estimates. It is reassuring that all scars, including BCG and unknown scars, were still strongly associated with better survival. Hence, the main result is not due to a peculiar misclassification of healthy individuals as having vaccinia scars rather than BCG or unknown.

Better survival among vaccinia-vaccinated individuals could be due to privileged groups being more likely to have received vaccination. In Bissau, vaccination was associated with entering secondary school, entering the military, international travels, and getting a job in the public sector [10]. Hence, more men than women were vaccinated according to the vaccination register of Bissau. However, controlling for these factors in the analysis had no impact on the estimated beneficial effect of vaccinia. In a remote rural setting, these selection factors are likely to have had much less impact as presumably most individuals were vaccinated in the occasional campaigns; for example, sex and years of schooling were not related to vaccinia scar prevalence in the study area. The coverage was highest in the villages near the two largest centres in which the campaigns were conducted in the colonial era (Table 1). The villages in-between might have had a lower coverage. Age was also associated with vaccination in the present study. Vaccinia coverage was lower among the youngest and the oldest, presumably certain age group were more likely to be called for vaccination in the campaigns. There may have been other determinants of vaccinia vaccination that we have no information about. For example, people sick or travelling on the day of vaccination campaign are unlikely to have received vaccinia subsequently. However, it seems unlikely that such factors would still play a role as confounding factors for survival 30–70 years later. Control for age, sex, and village did not modify the estimated beneficial effect of vaccinia vaccination.

The HIV-2 virus is endemic only in West-Africa. Few studies have examined the initial propagation of the HIV-2 virus in West-Africa. The epidemiology of HIV-2 is consistent with a period of more intense transmission in the 1950s or 1960s. A study from Guinea-Bissau suggested that the war of independence in 1963–74 may have been critical for the initial spread presumably due to the commercial sex and access to blood transfusions associated with the war [22]. The present and the previous study from Bissau city [10] suggest that smallpox vaccination campaigns in the same period may have been important as well, presumably due to insufficient sterilization of the knife or needle used for vaccination. Vaccinia vaccinations had the same beneficial effects among HIV-2 infected and HIV uninfected individuals.

Additional studies will have to be undertaken in other areas where selection biases may have been less or different or where original vaccination records are still available [23]. However, the larger arm-circumference among vaccinia-vaccinated adults may suggest that a biological process is involved. Furthermore, several case control and cohort studies from high-income countries have indicated that vaccinia vaccination may have had a protective effect against such diverse diseases as malignant melanoma [24], [25], rhabdomyosarcoma [26], Crohn's disease [27], asthma [23], and multiple sclerosis [28]. One study found better survival among malignant melanoma patients who had a vaccinia vaccination [29].

Our studies of vaccinia in Guinea-Bissau [10] and Denmark [22] were initiated prior to the recent interest in smallpox infection produced by bio-terrorism. Several previous studies had documented non-specific beneficial effects of live vaccines [3], [4], [6] and it was therefore logical to examine whether vaccinia had similar effects. So far the studies have supported that vaccinia may also have non-specific beneficial effects. If confirmed, it raises questions with major public health implications for low-income countries. Vaccinia induces long-term cellular immunity [30]–[32]. In animal models poxvirae may induce heterologous immunity, i.e. cross protection against other infections [33], [34]. Hence, it would seem important in humans to examine possible cross reactions between vaccinia responses and other infections as we might gain insight into the long term non-specific beneficial effects of this and other live vaccines. Vaccinia is very potent and now is regarded as having an unacceptably high incidence of side-effects, hence the use of modified vaccinia Ankara (MVA) as a vaccine and as a vector for other antigens [32]. It would seem important to examine whether MVA might have non-specific beneficial effects as well.
